# Concomitant stromal tumor and early cancer of the stomach: What should be done?

**DOI:** 10.1097/MD.0000000000007576

**Published:** 2017-07-21

**Authors:** Yan Xu, Liangfang Shen, Zhaoxia Lu, Xiaowei Liu, Wei Wu, Deyun Feng, Jaladanki N. Rao, Lan Xiao, Miao Ouyang

**Affiliations:** a Department of Oncology; b Department of Gastroenterology; c Department of Gastrointestinal Surgery; d Department of Pathology, Xiangya Hospital, Central South University, Changsha, Hunan, China; e Department of Surgery, University of Maryland School of Medicine, Baltimore, MD.

**Keywords:** concomitant, EGC, ESD, GST, outcomes, surgery

## Abstract

Concomitant gastric stromal tumor (GST) and gastric cancer (GC) is uncommon; even more uncommon is a concomitant GST and early stage GC (EGC). Tumor resection by endoscopic submucosal dissection (ESD) for concomitant GST and EGC has not been reported. We sought to define the clinical importance of detection of concomitant GST and EGC during the first esophagogastroduodenoscopy (EGD), and compare the clinical outcomes of ESD versus radical surgery for the treatment of concomitant GST and EGC. Our investigation was a retrospective cohort study. Patients with concomitant GST and EGC who underwent ESD or radical surgery were enrolled at the university-affiliated hospital from January 2005 to January 2015. The detection rate of concomitant GST and EGC during the first EGD was 3/25 (12%). Among 25 patients, 14 underwent ESD and 11 underwent surgery. Mean operation time and hospital stay were significantly shorter in the ESD group than the surgery group. There were no significant differences in terms of rates of en bloc resection, complete resection, and early complications. Late complications were more common in the surgery group than in the ESD group. The median follow-up duration was 58.9 months. Three- or 5-year overall survival rates were 100% for both groups and no patient died of EGC and GST. There was no local recurrence in the 2 groups; however, 3 metachronous EGC lesions were found during the follow-up period in the ESD group as follows: the simultaneous occurrence of GST and EGC was uncommon; the detection rate of concomitant GST and EGC was very low at the first EGD; and ESD appeared to be a safe, efficient, and popular treatment option for concomitant GST and EGC, that met the ESD absolute indication, and the outcomes were comparable to those achieved with surgery.

## 1 Introduction

Gastric stromal tumor (GST) and gastric cancer (GC) are common malignant tumors. GC is the fifth most common cancer and the third most common cause of cancer death worldwide.^[1,2]^ However, as the second most common cancer in the country, China has almost 50% of GC patients worldwide and most are found at advanced stages with a worse prognosis. Early GC (EGC) is defined as a neoplasm that is confined to the mucosa or submucosa, regardless of whether a regional lymph node metastasis is present.^[2,3]^ In a previous study, the rate of detection of EGC was about 10% to 20% in China, apparently much lower compared with 50% to 70%, or even more in Japan or Korea.^[4]^ Therefore, it is important that we improve the EGC detection rate, and the appropriate treatment plan for EGC patients.

Gastrointestinal stromal tumors (GISTs) comprise about 1% to 3% of all gastrointestinal tract malignant tumors, 50% to 70% of which are GST.^[5,6]^ In recent years, more GSTs have been found incidentally during routine esophagogastroduodenoscopy (EGD). GSTs have a malignant potential, particularly those originating from the muscularis propria layer. It has been demonstrated that 11% to 47% of GSTs have distant organ metastases at the first detection (at EGD, abdominal computed tomography [CT], etc.).^[7]^ According to the guidelines of the National Comprehensive Cancer Network, all GISTs > 2 cm should be resected. On the other hand, GISTs < 2 cm should either be resected or monitored. Sizes <5 cm can be removed by endoscopic or surgical resection.^[8]^

Endoscopic submucosal dissection (ESD) is an increasingly mature and advanced endoscopic technology for curative resection of early gastrointestinal carcinoma, precancerous lesions, and submucosal tumors. It is recently accepted as a standard treatment in patients with GST or EGC who have a negligible risk for lymph node metastasis.^[9,10]^ Many studies have demonstrated that ESD has high efficacy and safety in the treatment of GST or EGC, respectively. In addition, ESD for GST or EGC has shown long-term outcomes that are comparable to those of surgery.^[11–13]^ We used this technique to treat GIST or EGC in the past 8 years and produced positive therapeutic effects as well.

However, no published study has compared the clinical outcomes of ESD and surgical resection in patients with concomitant GST and EGC. We, therefore, conducted a retrospective cohort study comparing the therapeutic results of ESD with surgical resection to demonstrate the therapeutic usefulness of ESD for patients with concomitant GST and EGC within the absolute criteria.

The aim of our study was to evaluate the differences in the outcomes between the ESD and surgery groups, and to reemphasize the importance of increasing EGC detection rate.

## 2 Materials and methods

### 2.1 Patients

**Figure 1:**
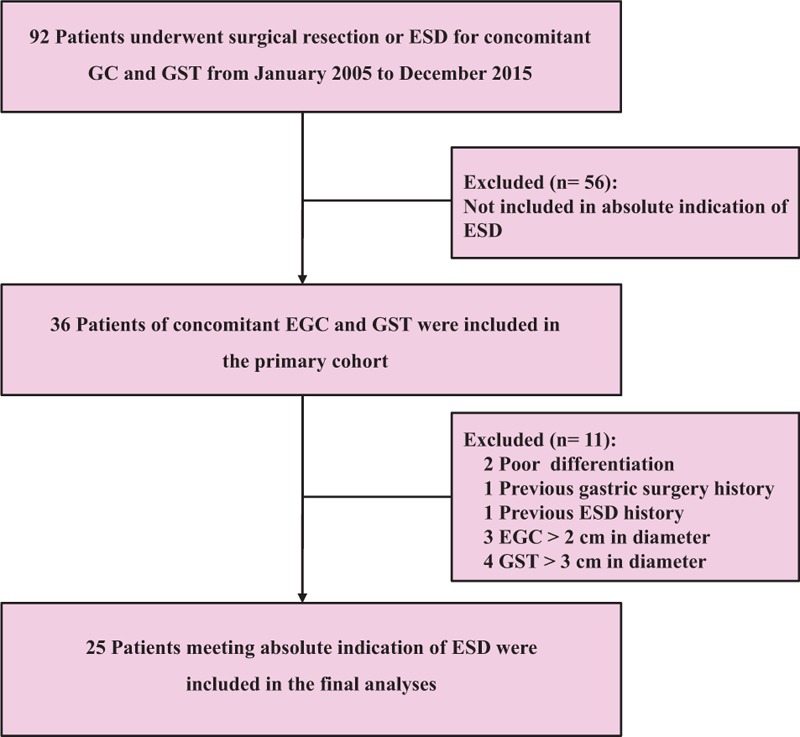
Flowchart of this study. ESD = endoscopic submucosal dissection, GC = gastric cancer, GST = gastric stromal tumor.

This was a retrospective study of 92 patients who underwent either surgical resection or ESD for concomitant GC and GST at the Xiangya Hospital, Central South University, Changsha between January 2005 and January 2015. The inclusion criteria, which met the absolute indications for ESD in concomitant GST and EGC, were as follows: no ulceration; a maximum GST of ≤3 cm, with no high risk endoscopic ultrasound (EUS) properties, such as irregular borders, cystic space, and heterogeneous echogenicity; ≤2 cm in diameter, of differentiated intramucosal adenocarcinomas for EGC; and no evidence of lymphovascular invasion, lymph node, and distant organ metastasis. Patients with a history of previous gastric partial resection, gastrectomy, or endoscopic resection were excluded. A total of 25 patients with concomitant GST and EGC were enrolled. Eleven patients had obtained surgical resection, while 14 patients had received ESD, since the ESD began in 2009 in the Xiangya Hospital. Figure 1 summarized a detailed flow chart of this study. Due to the single-centered and retrospective nature of the investigation, there was a possibility of selection bias; however, the majority of the data were collected in a systematic manner making the data relatively robust.

**Figure 2:**
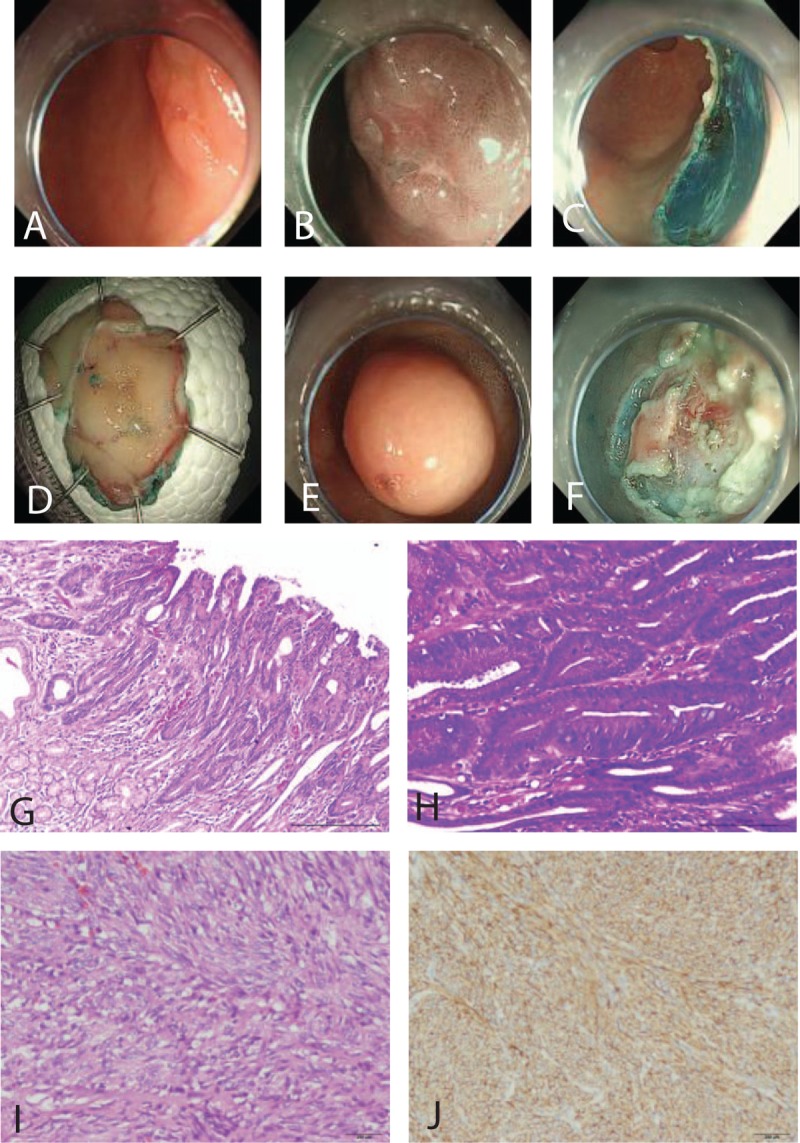
Endoscopic submucosal dissection (ESD) of concomitant early gastric cancer (EGC) and gastric stromal tumor (GST), pathological diagnoses. (A) EGC general endoscopic performance (located in gastric antrum posterior wall 12 × 18 mm). (B) Narrow band imaging + enlarged image of the same lesion. (C) Wound after ESD of EGC. (D) The completely resected EGC lesion with ESD. (E) GST (located in gastric fundus, 15 × 18 mm). (F) Wound after ESD of GST. (G) H&E stained, ×100 section shows high-grade intraepithelial neoplasia. (H) H&E stained, ×200 section shows local canceration, no lymphovascular invasion. (I) Typical photomicrograph of spindle cell gastrointestinal stromal tumor H&E, ×200. (J) c-kit (CD117) stained in the cytoplasm and cytoplasmic membranes, ×200. H&E = hematoxylin and eosin.

Before ESD and surgery, EGD, including narrow band imaging (NBI) staining and High-Definition amplification, was performed to confirm the locations of the lesions (Fig. 2A, B, and E), approximate size and surface morphology; EUS examination was performed to determine the size, depth of invasion, layer of origin, internal echogenicity, and growth pattern of GST and EGC; Abdominal CT scan with contrast was performed to determine growth pattern, tumor size, and to exclude possibility of lymph node and distant metastasis. A biopsy was performed when EGC was suspected during first EGD. The final diagnosis for the EGC or GIST was determined by histopathological evaluation after ESD or surgery.

Clinical features, such as baseline demographics, detection rate at the first EGD, tumor characteristics, operation time, hospital stay, complications, local recurrence, and overall and cause-specific survival were compared between the 2 groups.

The study protocol was approved by the ethics committee in our hospital, informed consent was obtained from all patients.

### 2.2 Endoscopic submucosal dissection and surgical resection

All procedures were performed under general anesthesia. Tracheal intubation was performed for mechanical ventilation.

A transparent cap (D-201-11802; Olympus, Tokyo, Japan) was attached to the end of the endoscope (GIF-Q260J; Olympus) and was used during the ESD procedure. The ESD procedure was performed as follows: first, the outside borders of the lesions, identified by conventional endoscopy or chromoendoscopy with NBI, were marked using dots. Marking dots were circumferentially placed, 2 (GST) or 5 mm (EGC) away from the margin of lesions with, argon plasma coagulation (APC; APC300, ERBE, Tübingen, Germany) probe; second, afterwards, 0.9% saline mixed with diluted epinephrine (1:100,000) and indigo carmine was injected submucosally around the lesion to lift it off the muscular layer. Third, the mucosa was incised circumferentially outside the marking dots with a dual knife (KD-650Q/U; Olympus). Fourth, a submucosal dissection was performed, with the IT-knife (KD-612L; Olympus) to allow complete removal of the lesion. If necessary during the procedure, the submucosal injection was repeated. Endoscopic hemostasis was achieved by hemostatic forceps (FD-410LR; Olympus) or hemoclips. Fifth, the artificial ulcer was cauterized with an APC to prevent delayed bleeding (Fig. 2C and F). Sixth, the incision was made wide enough to gradually expose and bulge out the body of the tumor. Then the submucosal tumor was either directly snared or dissected with IT knife when the root of the submucosal tumor was completely exposed. Following the tumor resection, clips were used to close the incision to prevent complications, such as bleeding or perforation. Tumor specimen was collected by using a stone basket or 3-claw forceps. Seventh, when perforation occurred, metal clips (HX-610-90, HX-610-135, Olympus; Resolution, Boston Scientific, Boston, MA) were used to occlude the perforation. The loop and clip technique was used when necessary.

Patients treated by surgery underwent an open gastrectomy with D1 or D2 or even more lymph node dissection.^[14]^ A subtotal gastrectomy or wedge resection was performed depending on the tumor location. Reconstruction methods included gastroduodenostomy or gastrojejunostomy, after distal gastrectomy. EGD was used intraoperatively to confirm tumor localization if necessary.

### 2.3 Histopathological evaluation

All resected specimens were flattened and stored in 10% formalin for pathological evaluation (Fig. 2D). GST samples were sectioned for pathological evaluation. Stained with hematoxylin and eosin (HE) (Fig. 2G, H, and I), immunohistochemistry analyses of CD117 (c-KIT) (Fig. 2J) and DOG-1 markers were performed to determine the nature of the tumor. Tumors that stained positive for CD117 (c-KIT) and DOG-1 were diagnosed as GST. The risk potential was determined in accordance with tumor size and mitotic index of the National Institutes of Health consensus risk classification. EGC specimens obtained from ESD were sliced serially at 2-mm interval,^[15,16]^ embedded in paraffin blocks, and stained with HE. Resected surgical specimens were prepared for pathological evaluation in a similar manner except that serial sectioning was performed at 5-mm intervals.^[15]^ According to the World Health Organization classification of GC, histological subtypes were classified as follows: histologically differentiated types included papillary adenocarcinoma and moderately or well differentiated tubular adenocarcinoma; undifferentiated types included signet ring cell carcinoma, mucinous adenocarcinoma, and poorly differentiated tubular adenocarcinoma.^[17]^

The resected specimens were evaluated for tumor involvement in the lateral and vertical margins, tumor size, depth of invasion, presence of ulceration, degree of differentiation, and lymphovascular invasion using a microscope.

### 2.4 Postoperative management and follow-up

Oral diet was suspended for about 3 days for all patients who underwent ESD or surgical resection, with GI decompression (1–3 days), and moved to a normal diet depending on the rate of improvement of symptoms. Proton pump inhibitors and prophylactic antibiotics were administered intravenously for 3 to 5 days, after which a proton pump inhibitor medication was orally taken for another 8 weeks.

EGD was performed 3, 6, and 12 months after resection, and then annually thereafter in all cases. To detect lymph node and distant metastasis, an abdominal CT and chest radiography were performed annually. In some patients, a positron emission tomography–CT was performed annually to evaluate the recurrence of GC and distant metastasis. A biopsy of the ESD scar tissue was performed at each EGD examination to evaluate the presence of local recurrence in patients who underwent ESD.

An adenoma or cancer found at a previous ESD site within 1 year was defined as “residual disease” and after more than 1 year as “local recurrence”. An adenoma or cancer found in the stomach at a different location other than the ESD site within 1 year was defined as a “synchronous lesion” and after 1 year as a “metachronous lesion”.^[36]^

### 2.5 Statistical analysis

Differences in patient characteristics and clinical pathological features between the ESD and surgery groups were evaluated by using the Student *t* test for continuous data, and the chi-square test or Fisher exact test for categorical variables. Continuous data were expressed as mean ± standard deviation. Factors associated with resectability, curability, and local tumor recurrences were analyzed using logistic regression. The survival rate was analyzed sing the Kaplan–Meier method and the log-rank test. Statistical calculations were conducted using SPSS version 19.0 for Windows software (SPSS, Chicago, IL), and *P* values <.05 were considered statistically significant.

## 3 Results

### 3.1 Baseline and clinicopathological characteristics

From January 2005 to January 2015, 25 patients with concomitant GST and EGC were enrolled in this study. All total of 14 patients underwent ESD in our endoscopy center and 11 patients underwent racial surgery in gastrointestinal surgery department of our hospital.

Patient and lesion characteristics are presented in Table 1. All features met the ESD absolute indication for EGC^[18]^ and GST (tumor size ≤3 cm).^[19]^ Horizontal or vertical resection margin involvements of the cancer and lymphovascular invasion were not found. There was no significant difference in age, gender distribution, comorbidity, tumor site, tumor size, tumor origin, histopathological characteristics, and proportion of patients with symptoms between the 2 groups (*P* > .05; Table 1). Most GSTs (80%, 20/25) were located in the gastric fundus and corpus, whereas most EGCs (88%, 22/25) where located in the gastric antrum and corpus; the mean follow-up duration was 49.8 months for the ESD group (12 cases >3 years and 10 cases >5 years). The mean follow-up duration was 68.1 months for the surgery group (only 1 case <3 years).

**Table 1:**
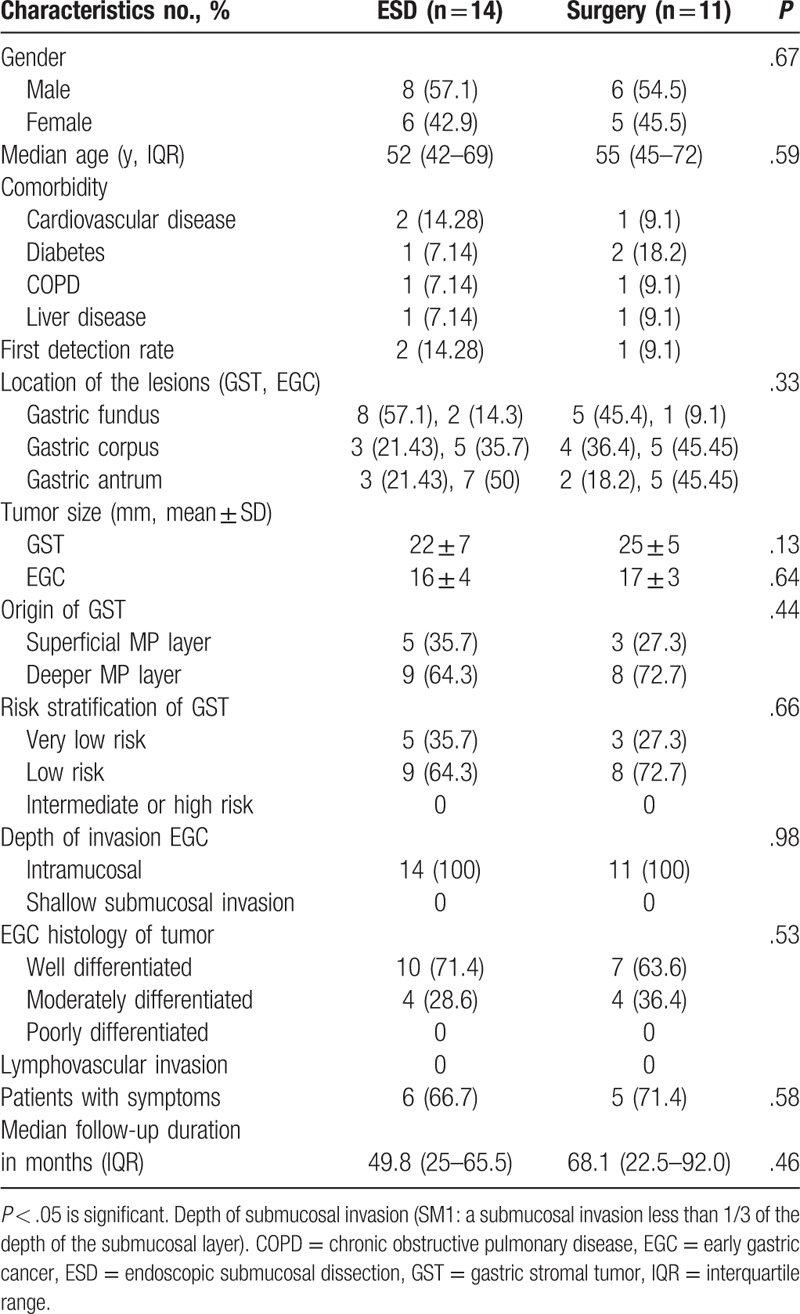
Baseline and clinicopathologic features of all patients (n = 25).

### 3.2 Detection rate at the first EGD

In the present study, among 25 patients, the detection rate of concomitant GST and EGC at the first EGD was 3/25 (12%) (Table 1). Presently, the EGC detection rate is about 10% to 20% in China. Therefore, our result could represent China's EGC detection level. Our result is significantly lower (*P* < .05) than that of Japan or Korea (50%–70% or more).^[4]^

### 3.3 Comparisons of short-term outcomes of ESD and surgery

Between the 2 groups, there were no significant differences, in terms of conversion to open surgery, tumor rupture, rate of an en bloc resection, complete resection, and early complication (Table 2). The ESD group had significantly shorter mean operation time (45.5 ± 13.5 vs 80.4 ± 22.7 min, *P* < .05) and shorter length of hospital stay (3.5 ± 1.5 vs 5.5 ± 2.7 days, *P* < .05) than the surgery group. Complete resection rate in both groups was 100%.

**Table 2:**
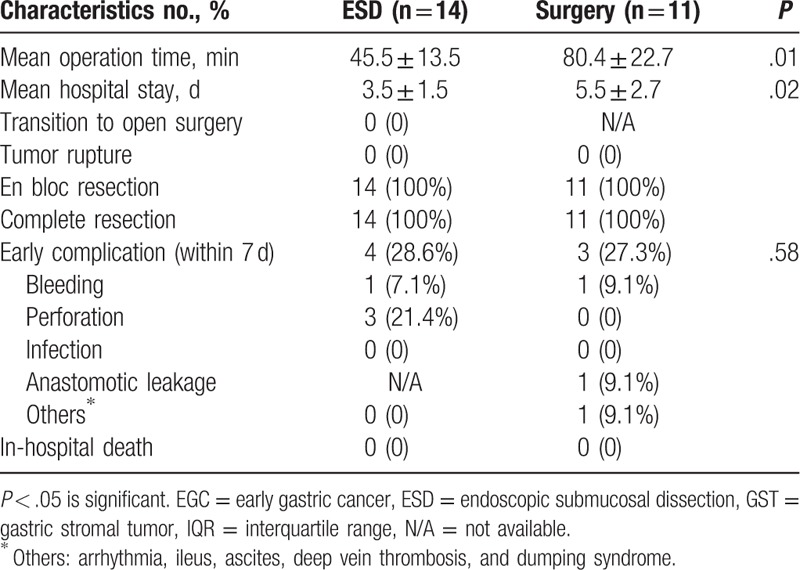
Comparison of short-term outcomes between ESD and surgery.

The overall early complication rate was not significantly different between the ESD group and surgery group (4/14, 28.5% vs 3/11, 27.3%, *P* > .05). The main early complications of ESD were perforation and bleeding. Perforations that occurred during the ESD procedure were found in 21.4% (3/14) and were managed successfully by endoscopic suture. Postprocedure bleeding within 7 days, occurred in 1 case each within the 2 groups and was managed successfully with endoscopic clipping or coagulation therapy, using a hot forceps. Anastomotic leakage and ileus occurred in 1 case of the surgery group, respectively, and was managed successfully by endoscopic clips and conservative treatment without additional surgery.

### 3.4 Comparisons of long-term outcomes of ESD and surgery

There were no hospital deaths, late perforation, infection, regional lymph node, and distant metastasis in either group during the follow-up period (Table 3).

**Table 3:**
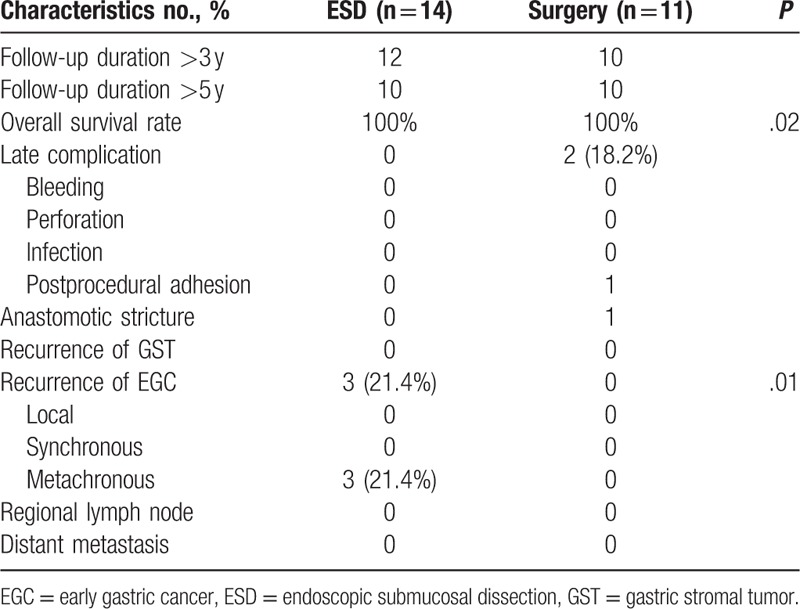
Comparison of long-term outcomes between ESD and surgery.

The late complication rate was 0% in the ESD group and 18.2% (2/11) in the surgery group (*P* < .05, Table 3). One patient from the surgery group, who experienced incomplete adhesive intestinal obstruction 3 years after surgery, was relieved with conservative treatment. Another patient from the same group experienced an anastigmatic stricture, but was successfully managed with an endoscopic treatment (balloon dilation and scar cut).

**Figure 3:**
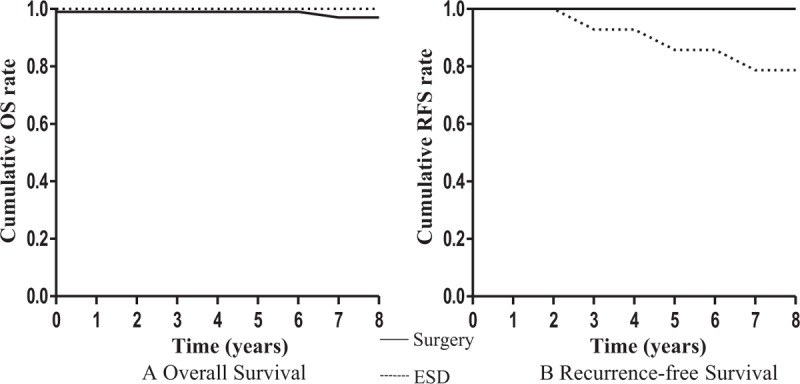
Log-rank test of long-term outcome of endoscopic submucosal dissection group and surgery group. (A) Overall survival, *P* = .893; (B) recurrence-free survival, *P* = .008.

The 3- or 5-year overall survival rates were 100% in both treatment groups (Fig. 3). There was no recurrence of GST in the 2 groups during the follow-up period. The 5-year GC recurrence rate in the ESD group was 21.3% (3/14), which was significantly higher than that of the surgical resection group at 0% (P < .05, Table 3). There was no local recurrence of a synchronous lesion (within 12 months) of primary EGC after ESD or surgery. Three cases of metachronous EGC (new lesion in different areas other than the first ESD site at least 1 year after the initial ESD or surgery, Fig. 3) were found in the ESD group (3/14, 21.3%), and successfully treated with additional ESD without affecting survival.

## 4 Discussion

Different histological types of GST and GC that arise in the same organ seriously endanger patients’ health. The prognosis of GST with concomitant GC depends primarily on the GC.^[20,21]^ However, the occurrence of this condition is uncommon. As the first case of concomitant epithelial and stromal tumors in the stomach was reported in 2000,^[22]^ more cases of concomitant GST and GC in the advanced stage of GC have been reported.^[23–25]^ GST with concomitant EGC has very low detection, with frequently missed diagnoses. Therefore, early detection and treatment of EGC is the key to improve survival of the patients.

In the present study, we retrospectively analyzed clinical characteristics of 25 patients with concomitant GST and EGC, and found the detection rate at the first EGD to be 3/25 (12%); most of the EGCs were missed. The remaining 22 patients were diagnosed by the second EGD detection before surgery. According to the more experienced doctors, the patients’ situations could not have been explained by GST alone. The result lies within the range of the present EGC detection rate in China (10%–20%). Several reasons could explain the low detection of EGC. First, GSTs (size >1 cm) are very conspicuous, and so many clinicians who lack the awareness of multiple primary tumors may have diagnosed GST alone, resulting in a low rate of preoperative diagnosis of EGC. Second, EGC lesions are small, and difficult to identify. In addition, the lesions are located in secluded portions of the stomach, such as the posterior wall of the gastric antrum, near the gastric corpus, the lesser curvature of the posterior wall of the gastric corpus, the gastric cardia, and others. In our study, most of EGCs (88%, 22/25) were located in the gastric antrum and corpus.

For the above reasons, we should ensure the following as endoscopy specialists: First, prepare adequately before an EGD inspection. Second, allow highly trained and experienced endoscopic doctors perform EGD examinations, to improve detection of the condition. Third, perform continuous screening of high-risk populations (aged over 40 years, high-prevalence area, *Helicobacter pylori* infection, gastric precancerous disease, GC family history, remnant stomach, etc.). Fourth, be unsatisfied with the discovery of 1 lesion, and pay close attention for the coexistence of 2 or more lesions, especially in patients with alarming symptoms, such as unexplained abdominal pain, weight loss, fluctuating of anemia and stool OB(+), and others. Fifth, focus on gastric mucosal changes under conventional endoscopy, including color and morphology; accurate biopsy with NBI staining and indicarmine staining combined with magnifying endoscopy should be utilized to obtain pathological diagnosis. These are feasible because at moment, high-definition and high-magnification endoscopy is widely available in urban and rural areas in China.^[4]^

The standard resection for EGC or GST is surgical gastrectomy with conventional lymph node dissection (open or laparoscopic); however, there are obvious disadvantages including more complications and significantly impaired quality of life. Since the development of endoscopic techniques in recent decades, ESD has been accepted as a popular treatment option for EGC or GST in China and Korea,^[26,27]^ whose features meet the ESD absolute indication even expanded indication. Several previous studies have compared the use of ESD and surgery for EGC or GST resection in different populations.^[28–30]^ Several multicenter retrospective studies and clinical experiences have suggested a satisfactory prognosis after ESD of high-grade dysplasia, early cancer or GST in the stomach, with results in high tumor eradication rates as well as a modality for the precise histological assessment of the entire lesion.^[31,32]^ Presently, no report exists for ESD in concomitant GST and EGC.

Patients who underwent ESD recorded shorter mean operation time and shorter hospital stay compared with the surgery group. In addition, our results revealed that there was no significant difference in tumor rupture, rates of en bloc resection, complete resection (100%), and early complications between both groups. Three- or 5-year overall survival rate were 100% in both treatment groups there were no GST and GC related death, nor were there lymph node and distant metastasis during the follow-up period. Therefore, the 5-year disease-specific survival rates were 100% too. Late adverse events, incomplete adhesive intestinal obstruction and anastomotic stricture occurred in the surgery group (2/11, 18.2%) only. Occurrence of metachronous EGC lesions was observed in the ESD group only (3/14, 21.3%). Similar to previous studies,^[33,34]^ the rate of metachronous GC is higher in our ESD group than the surgery group. The higher rate in the ESD group may be related to the larger remaining gastric mucosa area in the distal part of the stomach, which may have persistent helicobacter pylori infection^[35]^ and more severe glandular atrophy or intestinal metaplasia. However, our results were excellent and consistent with previous studies. All the complications and metachronous EGCs were successfully managed by endoscopic treatment (balloon dilation, scar cut, or additional ESD) and conservative treatment without impact on survival. Therefore, it is important to consider the occurrence of metachronous lesion. Aggressive and persistent endoscopic surveillance follow-up should be performed after ESD or surgical resection.

The pathogenesis of the synchronicity of GST and EGC has not yet been fully understood. Whether this is occasional, or there are potential mechanisms inducing the development of tumors of different histological types in the same organ,^[25]^ will be the focus of our future study.

Our present study has its own limitations. First, this study was a single-centered and retrospective investigation, suggesting potential selection bias; however, the majority of the data were collected in a systematic way making the data relatively robust. Second, the cohort group size was relatively small, due to the low detection rate of EGC in China, and low morbidity of the concomitant occurrence of GST and EGC. Large-scale multicenter, prospective, long-term follow-up studies will be necessary to confirm our findings in future.

In conclusion, our study revealed that ESD could be an effective first-line curative and popular management for concomitant GST and EGC that meet the ESD's absolute criteria for its comparable outcomes of surgical treatment. We should improve early detection rate of GC by developing physicians’ skills and increasing awareness as soon as possible.
